# miR-146a regulates glucose induced upregulation of inflammatory cytokines extracellular matrix proteins in the retina and kidney in diabetes

**DOI:** 10.1371/journal.pone.0173918

**Published:** 2017-03-16

**Authors:** Shali Chen, Biao Feng, Anu Alice Thomas, Subrata Chakrabarti

**Affiliations:** Dept. of Pathology and Laboratory Medicine, Western University, London ON, Canada; Cedars-Sinai Medical Center, UNITED STATES

## Abstract

Hyperglycemic damage to the endothelial cells (ECs) leads to increased synthesis of inflammatory cytokines. We have previously shown miR-146a downregulation in ECs and in the tissues of diabetic mice. Here we investigated the role of miR-146a, in the production of specific inflammatory cytokines and extracellular matrix (ECM) proteins in retina and kidneys in diabetes. We generated an endothelial specific miR-146a overexpressing transgenic mice (TG). We investigated these mice and wild type (WT) controls with or without streptozotocin (STZ) induced diabetes. Retinal and renal cortical tissues from the mice were examined for mRNAs for specific inflammatory markers, (ECM) proteins and inflammation inducible transcription factor by real time RT-PCR. Corresponding proteins, where possible, were examined using immunofluorescence or ELISA. In parallel, we examined ECs following incubation with various levels of glucose with or without miR-146a mimic transfection. In the retina and kidneys of WT mice with diabetes, increased expression of inflammatory markers (IL-6, TNFα, IL1β) in association augmented expression of ECM proteins (collagen 1αIV, fibronectin) and NF κB-P65 were observed, compared to WT non-diabetic controls. These changes were prevented in diabetic miR-146a TG mice along with retinal and renal functional and structural changes. In vitro studies showed similar changes in the ECs exposed to high glucose. Such changes were corrected in the cells following miR-146a mimic transfection. Further analyses of renal cortical tissues showed diabetes induced significant upregulation of two regulators of NFκB, namely Interleukin-1 associated Kinase 1 and tumour necrosis factor receptor associated factor. Such changes were prevented in diabetic TG animals. These data indicate that augmented production of inflammatory cytokines and ECM proteins in the retina and kidneys in diabetes are regulated through endothelium derived miR-146a. Identification of such novel mechanisms may potentially lead to the development of novel therapies.

## Introduction

Hyperglycemic damage to the endothelial cells (ECs) leads to increased synthesis of inflammatory cytokines and extracellular matrix (ECM) proteins [1]. Such glucose stimulated inflammatory cytokine and ECM protein production have been demonstrated in the context of diabetic retinopathy and nephropathy [2–4]. Structurally, mesangial matrix expansion and capillary basement thickening are two characteristic features of increased ECM protein production [5]. Several mechanisms are involved in such processes [6–8]. Several investigators have demonstrated NFκB activation due to sustained hyperglycemia [2,9–10]. NFκB acts as a key molecule in regulating gene transcription [11,12]. However, transcription factors require transcriptional co-activators for their action. P300 is a well-characterised transcriptional co activator. It acts through histone acetylation, making DNA accessible for transcription [13–15]. We have further shown that glucose induced upregulation of transcription co-activator p300 plays a vital role in regulating gene transcription in diabetes, through controlling activities of multiple transcription factors including NFκB [16–18]. In addition to regulation at the transcription level, post translationally, microRNAs (miRs) play key roles in synthesis of many cellular proteins [19–21].

miRs have received extensive attention in the recent years due to their regulatory roles in protein synthesis at the post-transcriptional and/or translational level; through which they control almost all biological processes [22–23]. They are possibly of importance in most of the diseases [24–26]. These ~22 nucleotide single-stranded RNA molecules mostly bind to 3’ UTR of specific target mRNA molecules and negatively regulate most gene expressions, either by translational repression or mRNA degradation [27,28].

We have previously shown that miR-146a is downregulated in the ECs and in the renal and retinal tissues of diabetic mice [29]. Other investigators have established its importance in inflammation [30–32]. It has been demonstrated that through targeting Interleukin-1 associated Kinase 1 (IRAK1) and tumour necrosis factor receptor associated factor 6 (TRAF6), it regulates NFκB activity [33]. Here we investigated the role of miR-146a, in the production of specific inflammatory cytokines and extracellular matrix (ECM) proteins in the context of diabetic retinopathy and nephropathy. Role of miR-146a (including miR-146a polymorphism) has been demonstrated in several chronic diseases. The list includes Alzheimer’s disease, renal ischemia reperfusion injury, coronary artery disease, inflammatory bowel disease, renal cell carcinoma and hepatocellular carcinoma [34–38]. miR-146a polymorphism has been shown to be a susceptibility factor in diabetic neuropathy [39]. We have recently also observed that miR-146a is downregulated in diabetic cardiomyopathy and it regulates expression of inflammatory molecules through such mechanism [40].

The purpose of this study was to examine whether miR-146a is altered in the retina and kidneys in a model of type1 diabetes and whether such alteration has any pathogenetic significance causing functional alterations in these organs. We examined such process at both *in vivo* and *in vitro*. As ECs are primary targets of tissue damage in diabetes, we used human retinal endothelial cells. In addition, to examine the role of endothelial derived miR-146a we used a unique tool. We generated an endothelial specific miR-146a overexpressing transgenic mice (TG). We investigated these mice and wild type (WT) controls with or without streptozotocin (STZ)-induced diabetes.

## Materials and methods

All chemicals and reagents were obtained from Sigma-Chemicals (St. Louis, MO, USA) unless otherwise specified.

### Cell culture

We used retinal endothelial cells as these are one of the main targets of diabetes-induced damage. Human retinal microvascular endothelial cells (HRECs, Cell Systems Corporation, Kirkland, WA) were grown in endothelial basal media-2 (EBM-2, Lonza, Walkersville, MD) containing supplements as described previously [1]. Cells were grown in 25 cm^2^ tissue culture flasks in humidified atmosphere containing 5% CO_2_ at 37°C. At 80% confluency, the cells were incubated in serum-free medium (without growth factors) overnight followed by treatments with various concentrations of glucose (5mM/L,normal glucose (NG); 25mM/L,high glucose (HG) or 25mM/L, L-glucose, osmotic control (LG)) at 48h timepoint.

### Animal studies

All animal experiments were performed in accordance with the Guiding Principles in the Care and Use of Animals. The protocols were reviewed and approved by the Western University Council on Animal Care Committee. The experiments were designed to conform with the National Institutes of Health *Guide for the Care and Use of Laboratory Animals* (publication no. 85–23, revised 1996).

In this study, we have used a transgenic mouse model with EC-specific overexpression of miR-146a generated by us, following the same method as described previously [41]. A cDNA fragment containing miR-146a was cloned into pg52pSPTg.T2FpAXK (pg52) plasmid comprising a Tie2 promoter, enhancer and a SV40 PolyA signal (courtesy of T. Sato, Nara Institute of Science and Technology Graduate School of Biological Sciences, Ikoma, Japan) [42]. The Tie2-miR-146a transgene was excised using *Sal*I, and this gel-purified transgene was injected into pronuclei of fertilized eggs C57BL/6 crossed with CBA/J mice. The oocytes were subsequently transferred into pseudopregnant females. The transgenic strains were identified by PCR-based assays of tail-tip genomic DNA specimens as previously described [41]. Three lines were generated and tested. Finally line #117 was used for subsequent studies. No basal phenotypic alterations were observed in the transgenic animals (data not shown).

Age-matched and sex-matched (males) miR-146a transgenic littermates were divided into diabetic and control groups. Diabetic groups were administered five intraperitoneal injections of STZ 50 mg/kg in citrate buffer (pH 4.5) (controls received the same volume of buffer) on consecutive days as previously described [43,44]. Blood glucose levels were measured 3 days after STZ injections to confirm hyperglycemia and the diabetic animals and controls were maintained for 2 months. Blood glucose was measured by Glucometer, Free Style Freedom Lite Inc, Alameda,CA, USA). Glycated hemoglobin was measured GlycoHb kit (Standbio Laboratories, TX,USA). The animals were sacrificed by deep anesthesia and exsanguination at the end of 2 months and renal cortical and retinal tissues and blood were collected for further studies.

### Isolation of bone marrow myeloid cells from mice

Femurs and tibiae were removed from 12 weeks old male TG mice and wild type controls. The bones were left in 70% ethanol for 2–5 min and washed with PBS. The ends were cut and the marrow flushed with PBS. Clusters within the marrow suspension were disintegrated by vigorous pipetting. Following PBS wash and centrifugation ~1–1.5×10^7^ leukocytes were obtained from each femur and tibia for miRNA analyses.

### RNA isolation and cDNA synthesis

Total RNA was extracted using Trizol reagent (Invitrogen, Burlington, Canada) as previously described [1]. Following quantification, 2 μg of RNA/sample were used for cDNA synthesis with high capacity cDNA reverse transcription kit (Applied Biosystems, Foster City, CA) and the cDNA generated were stored at -20°C.

### Real-time RT-PCR

Real-time RT-PCR was performed using the LightCycler (Roche Diagnostics Canada, Laval, Canada), as previously described [45]. mRNA levels were quantified by using the standard curve method. All data were normalized to 18S ribosomal RNA and/or β-actin.

### miRNA analysis

*mir*Vana miRNA Isolation Kit (Ambion, Austin, TX) was used to extract miRNA from tissues as described previously [41,43]. miRNA from serum was extracted using the miRNeasy Serum/Plasma Kit (QIAGEN, Toronto, ON) according to the manufacturer’s instructions. Real-time quantitative RT-PCR was performed with the TaqMan microRNA Assay in LightCycler 96 (Roche Diagnostic, Laval, QC) and data normalized to small nuclear RNA U6.

### miRNA mimic or antagomir treatments

HRECs were transfected with miRNA-146a mimics or antagomirs (Ambion, Invitrogen, CA) and scrambled controls (20 nmol/L) using Lipofectamine 2000 (Invitrogen, Canada). miRNA transfection efficiency was determined by real-time RT-PCR.

### Immunohistochemical and histochemical analyses

Formalin-fixed retinal and renal tissues embedded in paraffin were sectioned at 5 μm thickness on positively charged slides. The sections were stained with periodic acid-Schiff (PAS) and anti-mouse IgG antibody (1:400, MP Biomedicals, OH) as previously described [46]. IgG stained slides were scored based on the extravascular staining intensity (0–3).

### Western blotting and ELISA

Total tissue proteins were extracted and subjected to SDS gel electrophoresis (20 μg/sample), followed by Western blotting. The blots were incubated overnight at 4°C with rabbit IRAK-1, rabbit TRAF6, and rabbit NF-κB (1:200, Santa Cruz Biotechnology, Santa Cruz, CA) or mouse β-actin antibody (1:400) followed by 1h incubation with HRP-conjugated anti-rabbit antibody (1:5000, Santa Cruz Biotechnology, Santa Cruz, CA). Bands were visualized by chemiluminescence detection kit (Amersham Pharmacia Biotechnology, Buckinghamshire, UK) and quantified by densitometry [47].

Enzyme-linked immunosorbent assay (ELISA) was performed using a commercially available ELISA kits for Collagen IV alpha1 (Col 4α1) (Uscn Life Science Inc., Houston, TX), IL-1β and IL-6 (R & D Systems Inc., Minneapolis, MA) according to the manufacturer’s instructions.

### Urinary albumin and creatinine assessment

Urine samples were collected from diabetic and control animals placed in metabolic cages (24h) before sacrifice. Albumin/creatinine ratio (μg/mg) was measured using a commercial kit (Albuwell and Creatinine companion kit; Exocell, Philadelphia, PA). Assays were performed according to the manufacturer’s instructions.

### Statistical analysis

Data were presented as means ± standard error. Statistical significance of differences between groups was tested with Student’s *t* test or by using one-way ANOVA followed by post hoc analysis, as appropriate. A *p* value of 0.05 or less was considered to be significant and results are expressed as average of *n* = 6–8 animals per group.

## Results

### Glucose induces miR-146a downregulation in the endothelial cells

**Fig 1:**
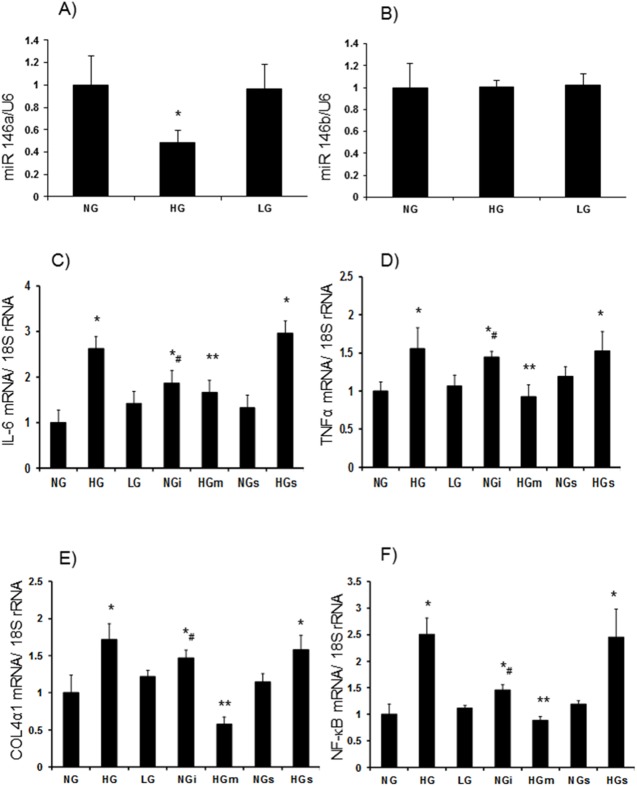
miR-146a expression analyses showed (*A*) reduced miR-146a in human retinal endothelial cells (HRECs) exposed to 25mM glucose (HG) compared to 5 mM glucose (NG). No alterations were seen in HRECs incubated with 25mM L-glucose (LG) or (*B*) miR146b expression levels in these conditions. *C-F*: HG caused upregulation of inflammatory markers (C) IL-6 and (D) TNFα, (E) ECM protein collagen 4α1 and (F) transcription factor NF-κB/p65 mRNA in the HRECs compared to NG. No such alterations were seen in LG. These upregulations were prevented following miR-146a mimics (HGm) transfection. Incubation of HRECs with miRNA antagomir (NGi) produced a glucose-like effect. [mRNA levels are expressed as a ratio to 18sRNA and miRNA expressions as a ratio of U6 snRNA (U6), both (mean±SE) normalized to NG s = scrambled RNA, *significantly different from NG, #significantly different from NGs, ** significantly different from HG or HGs, n = 5–6].

As outlined in our previous publications that, miR-146a is downregulated in the glucose exposed ECs and in the retina, kidney and heart in diabetes [29] and ECs are a main target of glucose-induced abnormalities, we first confirmed whether high glucose and diabetes caused downregulation of miR-146a in the endothelial cells. Following initial experiments at various durations (data not sown) and our previous reports [29, 43] we carried out these experiments after 48h of L-glucose incubation. Quantitative RT-PCR confirmed such downregulation in the retinal capillary endothelial cells following 48h exposure to 25mM glucose. No change was observed following 25mM L-glucose incubation. Furthermore, there were no changes in miR-146b Levels (Fig 1A and 1B).

### Glucose induced upregulation of inflammatory cytokines and ECM proteins are regulated by miR-146a

To gain an insight into the function of miR-146a, we focused on the inflammatory cytokines and ECM proteins, known targets of miR-146a [29,48–49]. To this extent we investigated interleukin 6 (IL-6), Tumour necrosis α (TNFα) collagen 4α1 (col4α1) and p65 component of key transcription factor NF-κB [50]. Twenty five mM glucose caused significant upregulation of TNFα, IL-6, Col4α1 as well as NFκB-p65. No changes were seen when the cells were exposed to L-glucose (osmotic control). To understand the direct role of miR-146a, we transfected the ECs exposed to high glucose with miR-146a mimic and ECs in low glucose with miR-146a antagomir; with scrambled miRs as controls. Although efficacy varied with respect to various molecules, glucose induced upregulation of these molecules were prevented by miR-146a mimic transfection. Furthermore transfection of miR antagomirs in the cells incubated in low glucose showed a glucose-like phenotypic change with respect to these molecules (Fig 1C, 1D, 1E and 1F).

### Diabetes induced upregulation of inflammatory cytokines and ECM proteins in the retinas and kidneys are regulated by miR-146a

We then expanded our study to establish whether such changes indeed happened in diabetic animals. For this purpose, we generated a unique tool, i.e, transgenic mice, with endothelial cell specific overexpression of miR-146a. We used a Tie2 promoter, kindly provided by Dr. Thomas Sato (Nara Institute of Science and Technology, Graduate School of Biological Sciences, Japan) [42]. The methods used were similar to transgenic (TG) mice we generated for miR200b and have been published previously [41]. As Tie2 is also present in the myeloid cells, we examined potential alterations of miR146a in the bone-marrow derived myeloid cells of the TG mice. No alteration of miR146a was seen in these cells in the TG mice (S1 Fig). Groups of TG and wild type (WT) littermate mice with STZ- induced diabetes were investigated and compared with age- and sex-matched non-diabetic controls. Diabetic animals showed low body weight, hyperglycemia, elevated glycated Hemoglobin levels and increased urine volume compared to non-diabetic animals. The TG animals showed no such phenotypic changes (S1 Table).

**Fig 2:**
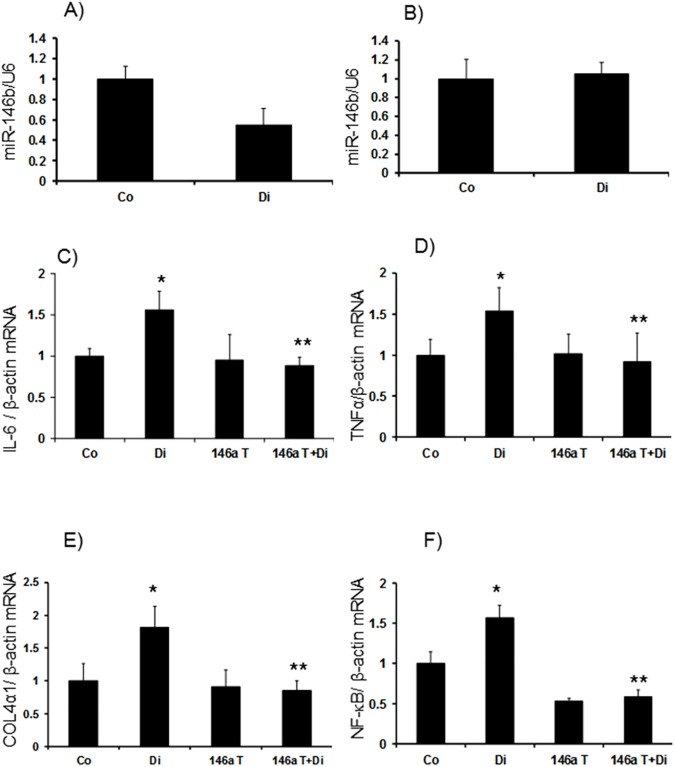
In the retinas of wild type animals with diabetes (Di), (*A*) miR-146a levels were reduced compared to controls (Co). Such reduction in levels was absent in (*B*) miR146b. mRNA analyses of the mice retinal tissues showed elevated mRNA expression of inflammatory markers (*C*) IL-6 and (*D*) TNFα, (*E*) ECM protein collagen 4α1 and (*F*) transcription factor NF-kB/p65 mRNA in wild type diabetic animals (Di) compared controls (Co). These changes were prevented in the retina of the diabetic mice with endothelial specific miR-146a overexpression (146aT+D: transgenic diabetic mice) [146aT: transgenic controls. mRNA levels are expressed as a ratio to β-actin and miRNA expressions as a ratio of U6 snRNA (U6), both (mean ± SE) normalized to Co, *significantly different from Co, **significantly different from Di, n = 6/group]

**Fig 3:**
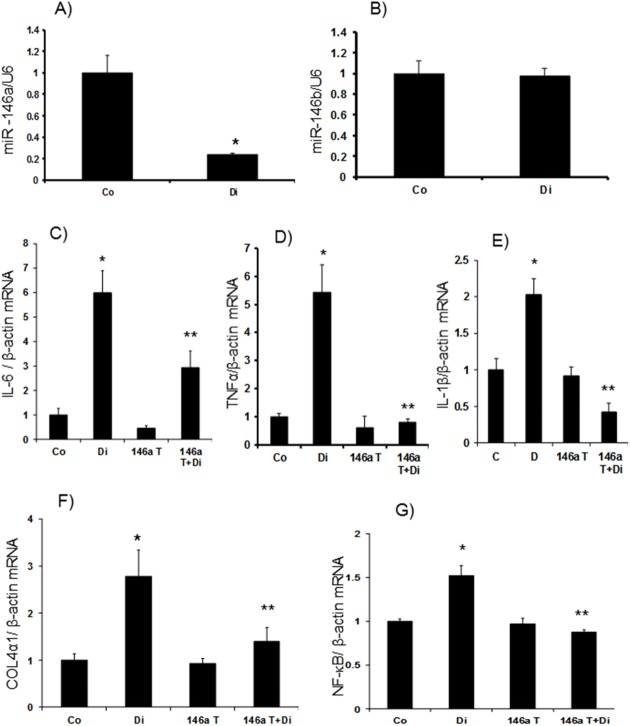
miR-146a expression in kidneys. (*A*) Expression of miR-146a was significantly lower in kidneys of wild type diabetic animals (Di) compared controls (Co). Similar reduction was not observed in expression levels of (*B*) miR-146b in the kidney tissues. mRNA levels of inflammatory markers (*C*) IL-6, (*D*) TNFα and (*E*) IL-1β, (*F*) ECM protein collagen 4α1 and (*G*) transcription factor NF-κB/p65 mRNA in Di compared to wild type controls. These changes were prevented in the kidney of the diabetic mice with endothelial specific miR-146a overexpression (146aT+D: transgenic diabetic mice) [146aT: transgenic controls. mRNA levels are expressed as a ratio to β-actin and miRNA expressions as a ratio of U6 snRNA (U6), both (mean ± SE) normalized to Co, *significantly different from Co, **significantly different from Di, n = 6/group]

**Fig 4:**
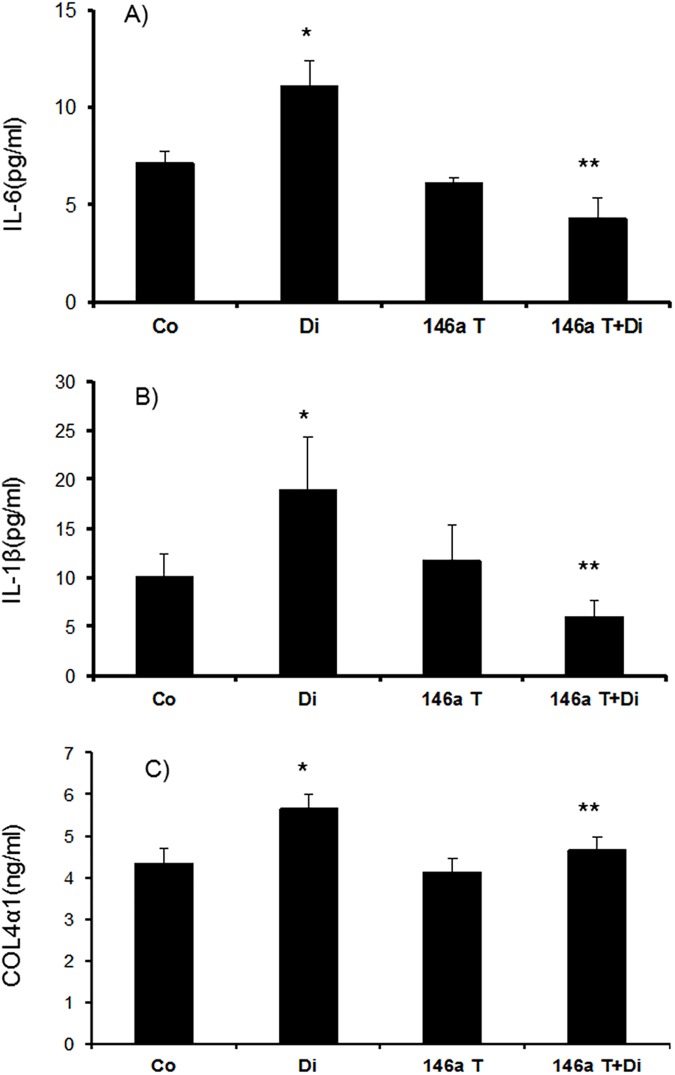
Protein analyses (mean ± SE) using ELISA of the mice renal tissues showed increased inflammatory markers (*A*) IL-6 and (*B*) IL-1β and (*C*) ECM protein collagen in diabetic animals (Di) compared to wild type control mice (Co). These changes were prevented in the kidney of the diabetic mice with endothelial specific miR-146a overexpression (146aT+D: transgenic diabetic mice) [146aT: transgenic controls, *significantly different from Co, **significantly different from Di, n = 6/group]

We then examined mRNA expression of IL-6 and TNFα. Both the retina and kidneys showed significant upregulation of these transcripts in diabetes. However, it is of interest to note that the levels of such upregulations were pronounced in the kidneys (~6 fold) compared to (~1.5 fold) the retina in diabetes (Figs 2C, 2D, 3C and 3D). In the kidneys, we examined another cytokine, IL1β, which also showed a twofold increase at the mRNA level (Fig 3E). As we had more material available in the kidneys we also performed protein analyses using ELISA for specific molecules. Both IL-6 and IL1β showed significant increase in diabetes (Fig 4A and 4B). All such changes were prevented in the kidneys and retinas of the transgenic mice with diabetes (Figs 2, 3 and 4).

We further examined ECM protein production in the retina and kidneys from these animals. Diabetes caused a~1.8 fold increase in Col4α1 mRNA production in the retina (Fig 2E). In the kidneys of diabetic mice Col4α1 mRNA was increased by 2.8 fold whereas Col4α1 protein was increased by ~1.5 fold (Figs 3F and 4C). All such changes were attenuated in the kidneys and retinas of the transgenic mice with diabetes (Figs 2, 3 and 4).

### MiR-146 upregulation prevents structural and functional changes in the kidneys and retina of diabetic animals

**Fig 5:**
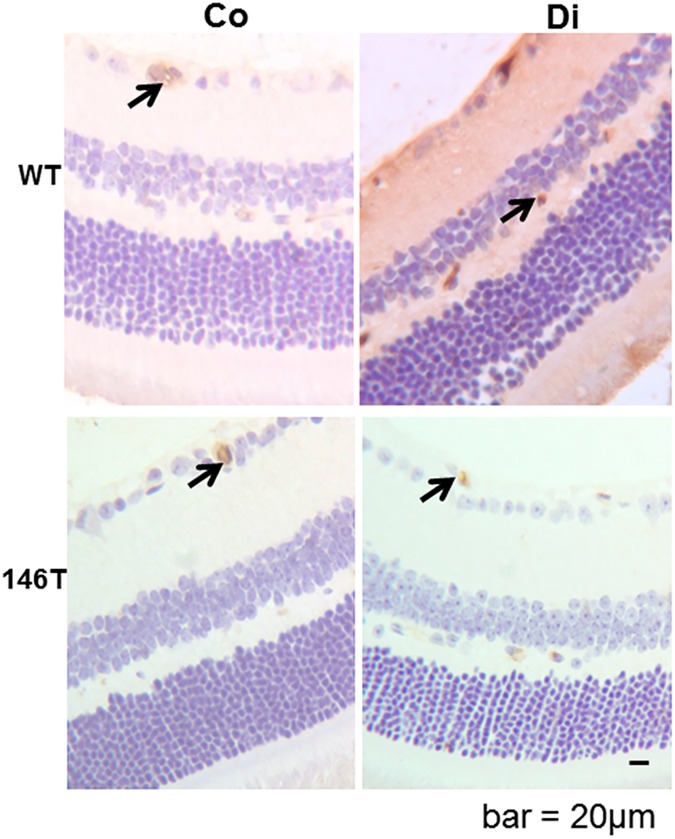
Altered functional changes in the retina due to diabetes and miR-146a overexpression. Immunohistochemical IgG staining of mouse retina showed increased extravascular diffuse stain, indicating increased extravasation (score 3, diffuse brown stain of whole retinal tissue) in the diabetic mice (Di) compared to wild type controls (Co) (score 0, brown stain limited to intravascular compartment). Increased extravasation was not observed in the transgenic mice with endothelial specific miR-146a overexpression (146aT: transgenic controls (score 0), 146aT+D: transgenic diabetic mice (score1)). [bar = 20um (same magnification for all micrographs), arrow = intravascular stain, n = 7 or more/group]

**Fig 6:**
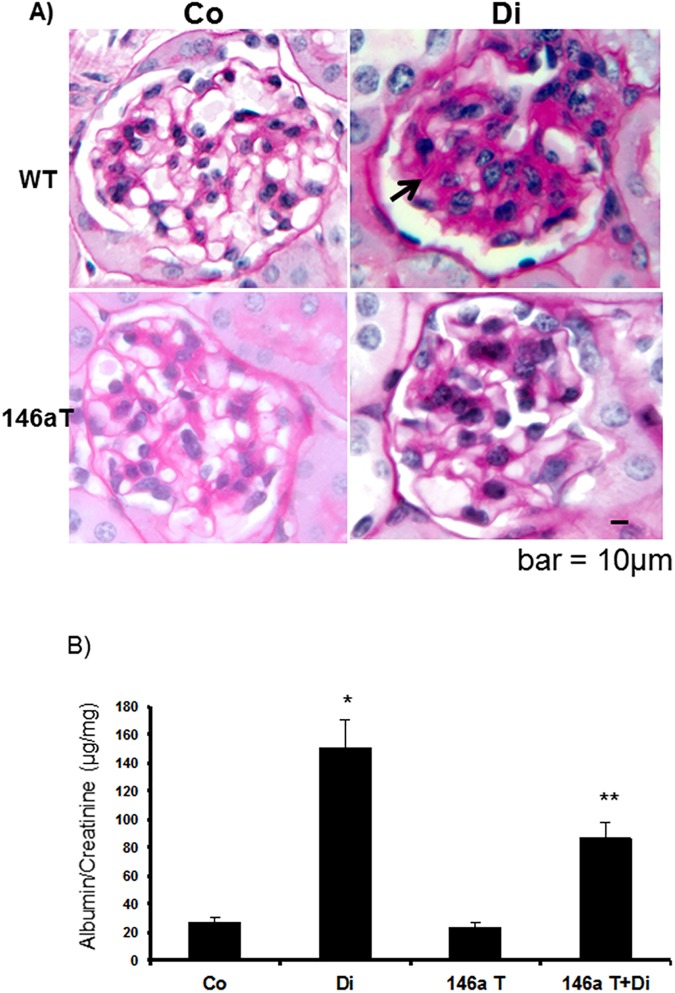
Modifications due to diabetes and miR-146a overexpression on the renal structure and function. (*A*) PAS stain showed mesangial expansion, indicating enhanced ECM protein deposition (arrow) in the glomerulus of diabetic (Di) animals compared to wild type controls (Co). Such diabetes-induced alteration in the miR-146a overexpression (146aT+D: transgenic diabetic mice). (B) In parallel, Di animals showed increased ACr ratio kidneys were prevented in the transgenic mice with endothelial specific, which was significantly reduced in the 146aT+D animals [146aT: transgenic controls, *significantly different from Co, **significantly different from Di. bar = 10um (same magnification for all micrographs) n = 7 or more/group]

We stained retinal tissues for vascular permeability alterations using immunoglobulin G (IgG) stain. This simple approach provides assessment of microvascular leakage. In the wild type diabetic mice, increased retinal stain for IgG, was indicative of increased vascular leakage. These alterations were prevented in the TG mice with diabetes (Fig 5). We further examined structural and functional changes in the kidneys. We investigated paraffin embedded renal tissue microscopically using PAS stain. In the wild type diabetic animals, PAS stain showed increased mesangial matrix deposition compared to diabetic animals. Such changes were prevented in the TG mice with diabetes (Fig 6A). In addition, we measured urinary albumin creatinine (ACr) ratio of these mice, from the urine collected at the time of sacrifice. Diabetes caused a significant increase in the ACr ratio. Such changes were corrected in the TG mice (Fig 6B).

### Mechanism of NF- κB activation by miR-146a in diabetes

**Fig 7:**
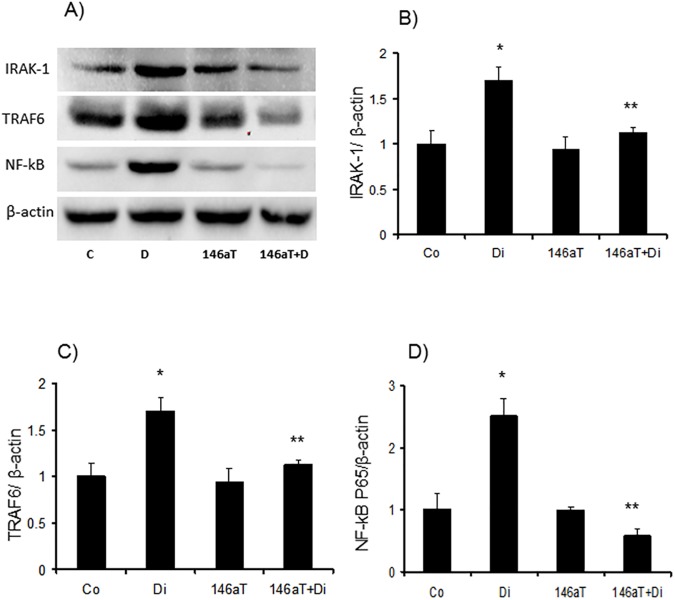
(*A*) Upregulation of miR-146a target proteins TRAF6 and IRAK1, were observed in the kidney tissue of wild type diabetic animals (Di) compared to controls (Co); in association with (*D*) increased NF-kB/p65 production. (*B*,*C*) quantitative analyses of the Western blots. This upregulation in target rpteons were prevented in the transgenic mice with endothelial specific miR-146a overexpression (146aT+D: transgenic diabetic mice) [146aT: transgenic controls, *significantly different from Co, **significantly different from Di, n = 6/group].

We performed these studies in the renal cortical tissues. Similar to *in vitro* experiments, we first examined possible alterations in NFκB-p65 levels at the tissue level. P65 mRNA and protein expressions were upregulated (1.5 fold and 2.5 folds respectively) in the renal cortex of the diabetic mice. Such changes were prevented in diabetic TG animals, indicating a miR-146a mediated regulation of these changes (Figs 3G, 7A and 7D). However, NFκB-p65 is not a direct target of miR-146a. Hence we examined other molecules which are direct targets of miR-146a and known to regulate NF-κB, namely TRAF6 and IRAK1. It has been shown that IRAK1 causes IL1-induced upregulation of NF-κB and TRAF6 is a known proinflammatory cytokines induced signal transducer [51]. Diabetes caused significant upregulation of these two transcripts. However, such alterations were prevented in the TG mice (Fig 7A, 7B and 7C).

## Discussion

In these experiments, we have shown that in the retina and kidneys, endothelium derived miR-146a plays a possible regulatory role on the expression of specific inflammatory cytokines and ECM proteins in diabetes. This process may contribute to the development of diabetes induced structural and functional changes and is mediated through NF-κB via IRAK1 and TRAF6.

Although miR146a has previously been investigated by us and others with respect to chronic diabetic complications [29,31,32,39], current study used some unique approaches to answer specific questions. This study was performed at multiple levels of complexities showing the effects of glucose and diabetes on augmented production of inflammatory cytokines and ECM proteins causing tissue damage. This study further delineates the mechanisms of such changes. In addition, we used a novel tool, an endothelium specific miR-146a overexpressing mice, which demonstrated that endothelial alteration of 146a is probably an initiating factor for these changes.

Increased expression of inflammatory cytokines and extracellular proteins are established changes in the organs affected by chronic diabetic complications [2–4]. ECs, being the first cell type exposed to high blood glucose levels, play a major role in the pathogenesis of chronic diabetic complications [3,29,52]. We carried out *in vivo* experiments in the retinal capillary ECs. It is also to be noted that glucose transport in these ECs are insulin-independent [53]. Hence in hyperglycemia, intracellular glucose level rises, causing increased glycolysis, activation of multiple metabolic abnormalities and increased mitochondrial superoxide generation [54]. Ultimately, cellular transcription machinery gets activated and augmented production of inflammatory cytokines and ECM protein ensue [2–4]. At the post transcription levels, such processes are regulated by miRs [29,41,43,55].

We have previously shown that miR146a is reduced in the retina and kidneys because of sustained hyperglycemia [29]. On the other hand, in diabetes, augmented expression of miR-146a has been demonstrated in the cornea [56]. In keeping with our findings, a recent publication however, has demonstrated a protective role of miR146a on diabetic nephropathy [57]. It is also interesting to note that, although some investigators have demonstrated glucose induced reduced miR-146a in the retinal endothelial cells, others have demonstrated increased miR146a in the umbilical vein endothelial cells following short term glucose exposure [58, 59]. These findings suggest that in diabetes, tissue specific and duration dependent variation exists with regards to miR146a expression. Here, we have shown that reduced miR-146a activates pro-inflammatory transcription factor NF-κB through it’s targets, namely IRAK1 and TRAF6, ultimately causing increased inflammatory cytokine and ECM protein production causing structural and functional changes in the tissues.

To characterise these changes, we have developed and used a unique tool, TG mice with endothelial specific overexpression of miR-146a. Using such approach, we have previously shown the role of miR-200b in the pathogenesis of chronic diabetic complications [41]. We have also shown similar effect of miR-146a in the heart in diabetes, another organ affected by chronic diabetic complication [29,40]. It is of interest to note that the effect of miR-146a on inflammatory cytokine production via NF-κB is not direct but mediated indirectly through lRAK1 and TRAF6. Similar mechanisms have been shown in other systems [33].

Nevertheless, data from this study opens up the possibility of novel RNA-based treatment approach, targeting miRs. However, such approaches are challenging as single miRs may regulate multiple mRNAs and one mRNA can be regulated by multiple miRs [21]. Hence “off-target” effects of miRs from a therapeutic standpoint remain a critical issue. On the other hand RNA-based therapies are attractive due to their specificity [60] The list of altered miRs in chronic diabetic complications include miR-200b, miR-155, miR-1, miR-320, miR-195 among others [28,43,60,61]; each targeting various RNA molecules. We believe that all of these are of importance, controlling various pieces of this puzzle. Similarly other non-investigated miRs and other pathogenetic mechanisms may also exist. It is further worth mentioning that other epigenetic mechanisms including other non-coding RNAs, may regulate specific miRs. Additionally, miRs may regulate other key molecules mediating specific epigenetic functions [55]. Hence, a complex web of intricate regulatory mechanisms exist which need further clarification and further investigations are necessary to decipher these mechanism and identification of treatment targets.

Characterization of these mechanisms using TG mice with EC specific overexpression of miR-146a, further suggests a role of ECs in the initiation of chronic diabetic complications. ECs possibly are the primary targets of hyperglycemic damage [16,62]. In addition, direct effects of glucose on other cells possibly further augment such damage. EC derived factors may influence other cells, ultimately causing damage to the whole organ and impair organ functions. Hence at the cellular level, endothelial cells could lend themselves as a target cell for the prevention and treatment of chronic diabetic complications.

In summary, we have demonstrated at multiple levels of complexities in the role of miR146a in the pathogenesis of diabetic retinopathy and nephropathy. However, we do recognise that in these chronic diseases other important mechanisms, including other miRNAs, possibly also have significant pathogenetic roles. More in depth analyses of functional consequences remains to be performed to understand such processes and to develop potential future RNA based therapeutics.

## Supporting information

S1 Fig miR-146a expression analyses (mean ± SE) in the isolated myeloid cells showed no significant alterations of the miR-146a levels in the myeloid cells of the transgenic mice (146aT) compared to wild type controls (WT). miRNA levels are expressed as a ratio of U6 snRNA (U6), n = 6/group].
(TIF)

S1 Table Results from clinical monitoring of the mice. (DOC)
