# Supplementary figures and images for: miR-146a regulates glucose induced upregulation of inflammatory cytokines extracellular matrix proteins in the retina and kidney in diabetes
Source: PLoS One. 2017 Mar 16;12(3):e0173918. doi: 10.1371/journal.pone.0173918 (PMC5354466; doi:10.1371/journal.pone.0173918)

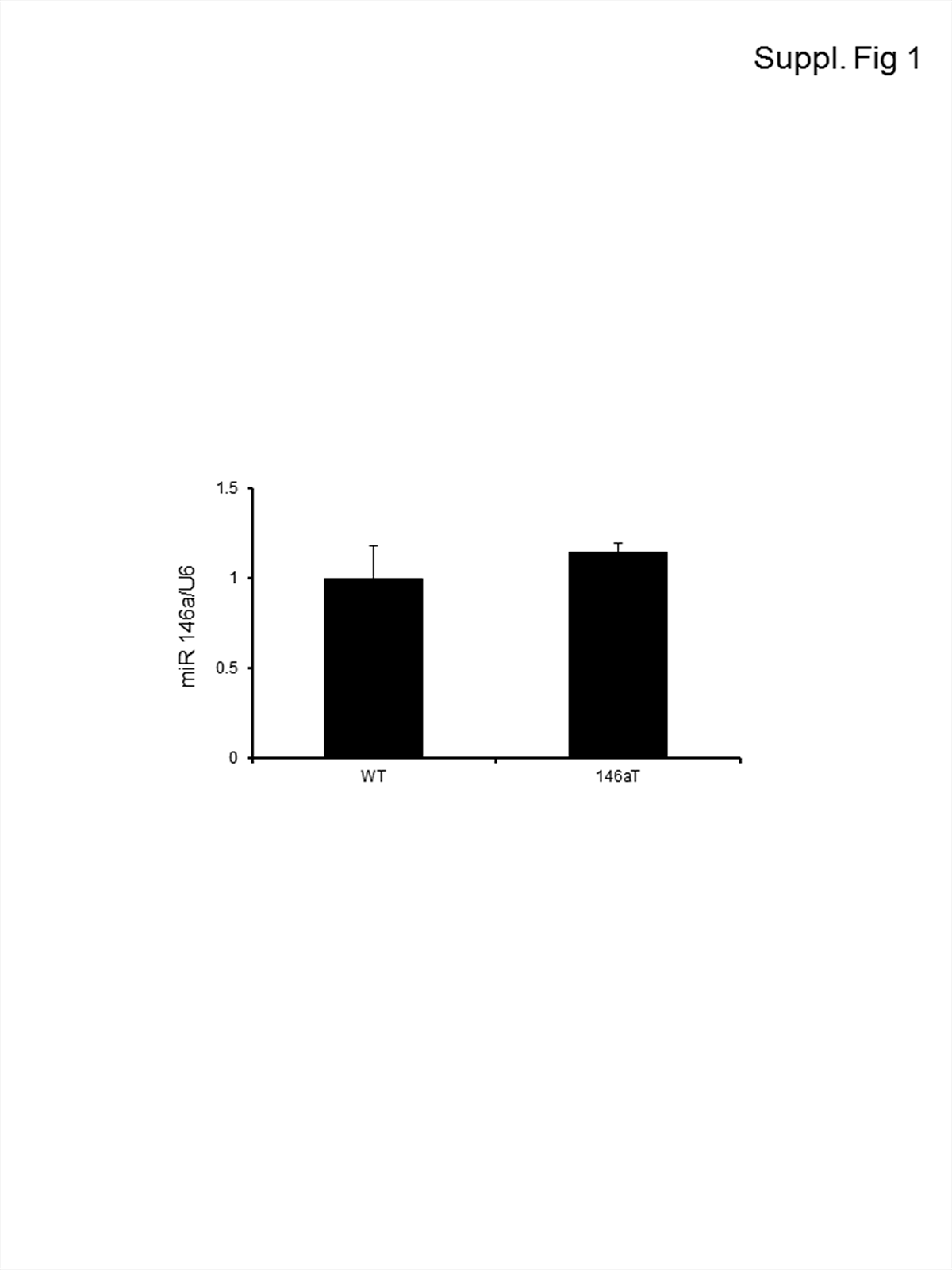

Supplement: S1 Fig — miRNA levels are expressed as a ratio of U6 snRNA (U6), n = 6/group]. (TIF) [file pone.0173918.s001.tif]
