# Supplementary material for: miR-146a regulates glucose induced upregulation of inflammatory cytokines extracellular matrix proteins in the retina and kidney in diabetes
Source: PLoS One. 2017 Mar 16;12(3):e0173918. doi: 10.1371/journal.pone.0173918 (PMC5354466; doi:10.1371/journal.pone.0173918)
Supplement: S1 Table — (DOC) [file pone.0173918.s002.doc]

**Suppl. Table 1**: Clinical Monitoring

| Groups | Body Weight (g) | Blood Glucose (mmol/L) | HbA1c (%) |
| --- | --- | --- | --- |
| B6-Control | 29.50±1.72 | 7.27±0.87 | 4.21±0.18 |
| B6-Diabetic | 22.58±1.62* | 24.48±2.58* | 7.96±0.31* |
| miR-146a-Control | 30.82±1.17 | 7.57±1.28 | 4.26±0.19 |
| miR-146a-Diabetic | 23.20±3.05† | 25.13±2.75† | 8.03±1.22† |

Note: n=11 to 13/group, [B6= wild type, miR-146a = miR-146a transgenic mice, * *p* < 0.05

compared to B6 control group, † *p* < 0.05 compared to 146a-control group]
